# Immunomodulatory and Anti-Inflammatory Properties of Selenium-Containing Agents: Their Role in the Regulation of Defense Mechanisms against COVID-19

**DOI:** 10.3390/ijms23042360

**Published:** 2022-02-21

**Authors:** Valentina N. Mal’tseva, Michael V. Goltyaev, Egor A. Turovsky, Elena G. Varlamova

**Affiliations:** Institute of Cell Biophysics of the Russian Academy of Sciences, Federal Research Center “Pushchino Scientific Center for Biological Research of the Russian Academy of Sciences”, 142290 Pushchino, Russia; maltsevavn@gmail.com (V.N.M.); goltayev@mail.ru (M.V.G.)

**Keywords:** selenium, selenoproteins, selenium nanoparticles, sodium selenite, methylseleninic acid, selenomethionine, methylselenocysteine, immune system, COVID-19

## Abstract

The review presents the latest data on the role of selenium-containing agents in the regulation of diseases of the immune system. We mainly considered the contributions of selenium-containing compounds such as sodium selenite, methylseleninic acid, selenomethionine, and methylselenocysteine, as well as selenoproteins and selenium nanoparticles in the regulation of defense mechanisms against various viral infections, including coronavirus infection (COVID-19). A complete description of the available data for each of the above selenium compounds and the mechanisms underlying the regulation of immune processes with the active participation of these selenium agents, as well as their therapeutic and pharmacological potential, is presented. The main purpose of this review is to systematize the available information, supplemented by data obtained in our laboratory, on the important role of selenium compounds in all of these processes. In addition, the presented information makes it possible to understand the key differences in the mechanisms of action of these compounds, depending on their chemical and physical properties, which is important for obtaining a holistic picture and prospects for creating drugs based on them.

## 1. Introduction

**Figure 1:**
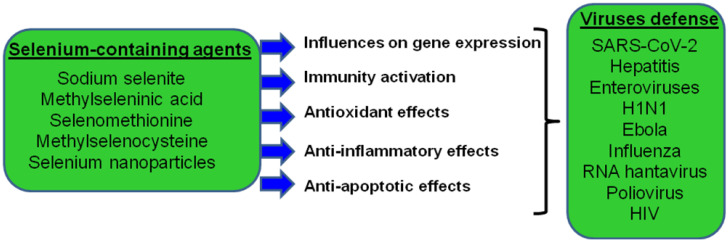
Schematic representation of the main pathways of antiviral action of selenium-containing agents. Protective effects refer to virus-infected cells.

The immune system provides protection against pathogens and maintains tissue homeostasis throughout the lifetime of the body. Examples of immune systems are found in multicellular organisms as simple and ancient as sea sponges. To date, enough information has been accumulated indicating the involvement of microelements selenium (Se) and various selenium-containing compounds in the regulation of the immune response (Figure 1) and oxidative stress [1,2,3,4,5,6,7]. According to the World Health Organization (WHO), the Se consumption rate is as follows: for adult women, 55 µg/day, and for adult men, 70 µg/day. According to various sources, the upper permissible level of Se consumption can range from 300 to 600 μg/day. The toxic dose is considered to be 900 μg/day [6,8,9]. There is a problem of dietary Se deficiency in some regions of China—Se deficiency in conjunction with viral infections leads to Keshan disease and cardiomyopathy [10,11]. In some areas of Siberia and Tibet, Se deficiency leads to male infertility and to the development of Kashin−Beck disease [10,11,12,13]. However, in countries where the population does not experience Se deficiency, there are groups of people characterized by a reduced level of Se—vegetarians, people on long-term kidney hemodialysis, people with HIV infection, and those with impaired thyroid function [10,14,15,16,17]. Dietary and supplemental Se has been shown to improve treatment prospects for some cancers in oncology [18,19], although more detailed studies are needed due to the available data on toxic doses of Se. However, in the case of cardiovascular disease in Europe and the USA in patients receiving sufficient dietary Se, the supplementation of this trace element in the form of dietary supplements does not affect the risk of stroke [20,21]. Acute Se toxicity resulting from a chronically high Se intake (regular use of brazil nuts and some over-the-counter drugs) can cause severe gastrointestinal and neurological symptoms, acute respiratory distress syndrome, myocardial infarction, hair loss, muscle soreness, tremor, dizziness, facial flushing, kidney failure, heart failure, and, in rare cases, death [10,22]. The effects of additional Se intake to stimulate the immune system and protect against viral infections require more research, but a clear correlation has already been shown for a high percentage of COVID-19 infections in Suizhou and Xiaogan, Se-deficient regions of China, compared with Enshi, Yichang, and Xiangyan, where the dietary Se intake is even slightly higher than normal [23,24]. Elsewhere in the world, in South Korea, India, Iran, and Russia, patients with severe COVID-19 have also been found to have reduced plasma Se levels, and these same people are deficient in their dietary Se intake [25,26,27,28]. In European countries, like Germany and Belgium, where the population is not deficient in dietary Se, severe COVID-19 disease has also been shown in patients with low levels of this trace element and its SELENOP carrier protein in the plasma [29,30].

The importance of adequate dietary Se levels and its effective incorporation into selenoproteins for immunity has been demonstrated in cell culture models, rodent models, livestock and poultry studies, and humans. Se deficiency can cause immune deficiency, leading to an increased susceptibility to infections [31,32,33,34,35,36,37], diabetes mellitus [38], Keshan disease [39,40], and possibly cancer [41,42]. Selenium deficiency and the suppression of selenoprotein expression are associated with higher levels of inflammatory cytokines in various tissues, including the gastrointestinal tract, uterus, and breast tissue [43,44,45,46,47]. However, some inflammatory processes are actually intensified when the Se intake changes from insufficient to sufficient. For example, a mouse model of allergic asthma has shown that Se deficiency reduced airway inflammation, while adequate Se intake resulted in higher levels of inflammation, which were then reduced with the use of abnormal Se levels [48].

In addition, it is well known that Se is involved in the regulation of defense mechanisms against various viral diseases. Viral infections are a serious public health problem in many parts of the world due to their widespread prevalence, the large number of pathogenic viruses, and significant socio-economic damage, especially due to the recently emerging coronavirus disease, COVID-19. Understanding the mechanisms of infection of the body with viruses, the characteristics of the course of the disease and the relationship with certain vital microelements supplied with food will contribute to the development of more effective ways to resist viral infections.

Selenium deficiency leads to an increase in the body’s sensitivity to a number of viral infections due to the influence on various intracellular mechanisms of both the virus itself and the host cell. Selenium affects the mechanism of attachment of a viral particle to the host cell membrane, probably due to the inhibition of disulfide exchange reactions, which leads to a decrease in the expression of disulfide proteins in the viral glycoprotein, disclosure of hydrophobic epitopes, and inactivation of the ability of the virus to cross the bilayer membrane of the host cell [49].

Thus, it can be said that the high content of Se in the body assists in the prevention of viral diseases. For example, for the human immunodeficiency virus (HIV), a clear correlation between the Se content in the body and the course of the disease has shown that an increased level of this microelement helps to alleviate the symptoms and consequences of the disease and the degree of spread of HIV type 1 by stimulating the activity of glutathione peroxidase [50]. A similar antiviral mechanism of exogenous Se has been shown for people infected with hepatitis B and C viruses [2]. In regions of China with a low Se status, a disease such as hemorrhagic fever with renal syndrome caused by RNA *Hantaviruses* is six times more common. This disease at the molecular level is accompanied by the suppression of the expression and activity of the selenoprotein glutathione peroxidase 3 (GPX3). At the same time, studies on HUVEC cells have shown that the addition of Se to the culture medium significantly reduces the degree of infection of these cells with the hantaviruse virus [51]. The positive effect of dietary Se compounds has been shown in people infected with poliovirus, when an increase in the concentration of Se in the blood promoted the elimination of viral particles from the body and suppressed the ability of the virus to mutate [52]. In this study, it was shown that a low Se status contributed to the emergence of serious mutations during infection with coxsackievirus B3 and influenza virus [38].

It is well known that Se modulates the signaling functions of many regulatory proteins, providing beneficial effects in inflammatory diseases [53]. A prime example of chronic inflammatory disease is endometritis, which interferes with reproductive function in both humans and animals. The main pathogen causing endometritis is *Staphylococcus aureus*, the Gram-negative bacillus, which is the causative agent of many invasive infections. This pathogen is recognized by TLR2 (Toll-like receptor 2) and other members of the TLR family, which are evolutionarily conserved receptors for cells of the innate immune system. When signaling from TLR2, the NF-κB pathway is activated, which leads to the synthesis of pro-inflammatory cytokines and chemokines after the recognition of microbial components or ligands formed at the sites of infection.

The main emphasis in this review will be placed on the very urgent and dangerous respiratory disease caused by Severe Acute Respiratory Syndrome coronavirus 2 (SARS-CoV-2), COVID-19. The syndromes of this disease are associated with the release of a large amount of pro-inflammatory cytokines, inflammation, and increased ROS (reactive oxygen species) production, which lead to acute lung damage, hypercoagulability, and multiple organ failure [54]. The work will provide all the information available at the moment on the participation of the most common selenium-containing agents of an organic and inorganic nature, as well as nanoparticles of Se and selenoproteins in the regulation of immunomodulatory, anti-inflammatory, and antiviral functions. In addition, the key mechanisms of such regulation will be considered, as well as the latest data on the involvement of Se-containing agents in the prevention and treatment of COVID-19.

## 2. Participation of Sodium Selenite in the Regulation of Diseases of the Immune System of Various Etiologies: Possible Role of Sodium Selenite in the Treatment of COVID-19

**Figure 2:**
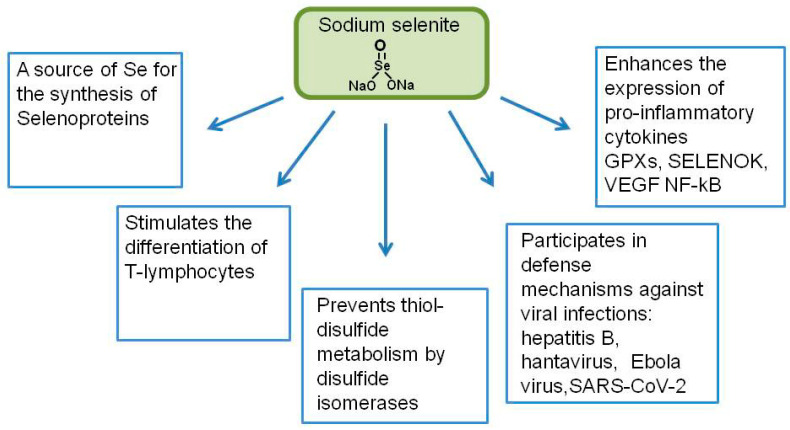
Participation of sodium selenite in the regulation of the gene expression and activation of the immune system. GPXs—glutathione peroxidases; NF-κB—nuclear factor kappa-light-chain-enhancer of activated B cells; SELENOK—selenoprotein K; VEGF—vascular endothelial growth factor.

Among inorganic Se compounds, the most widespread and those used for medical purposes are selenites, in particular, sodium selenite (Figure 2). The metabolism of SS is well studied: it is reduced by thioredoxin reductase (TrxR), and glutathione (GSH), and also by the systems of thioredoxin (Trx) and glutaredoxin (Grx) to selenodiglutathione (GSSeSG). Subsequently, GSSeSG is reduced by the same systems via selenopersulfide to hydrogen selenide (H_2_Se), the key metabolite of Se in the cell [55,56,57,58]. Hydrogen selenide is involved in many reactions in the cell, in particular, it is metabolized to methylselenol (CH_3_SeH) and later to dimethylselenide ((CH_3_)_2_Se), which is excreted from the body with respiration, and trimethylselenonium ((CH_3_)_3_Se)^+^, which is excreted in the urine [59]. Hydrogen selenide, which is formed from selenites, is involved both in the synthesis of selenosaccharides (excretion) and in the synthesis of methylselenol and selenoproteins. In addition, SS (Sodium selenite) is able to directly interact with the sulfhydryl groups of cell membranes, converting them into disulfide groups, while SS itself is reduced to elemental Se with a characteristic red color [60,61].

Among the mechanisms of antioxidant and anti-inflammatory action of SS, as a rule, the following are distinguished: it is a source of Se for the synthesis of selenoproteins, which have antioxidant, antitumor, anti-inflammatory, and other activities; it affects the regulation of the activity of cytokines and various antioxidants; it directly affects the cells of the immune system (stimulation of T-lymphocyte differentiation and NK-cell activity); and it prevents thiol/disulfide metabolism using protein disulfide isomerases (there is a decrease in the ability of the viral spike proteins to interact with ACE2) [62,63].

A study of SS exposure performed on human hepatoma cell lines, HepG2 and Huh7, showed that the observed increase in the expression of selenoproteins GPX1 and SELENOK is correlated with an increase in SS concentration, and vascular endothelial growth factor (VEGF) and three pro-inflammatory cytokines (IL-6, IL-8, and IL-17) decreased with the increasing selenite concentration. These data may indicate the anti-inflammatory function of SS [64]. In addition, it has been shown that SS is a non-toxic anti-inflammatory agent that reduces the lymphedema volume [65]. The potential anti-inflammatory effect of SS has been shown in a mouse model of asthma. The pre-application of SS to the peritoneum of allergen-sensitive (ovalbumin) mice resulted in a decrease in the negative effects of ovalbumin: activation of the transcription factor NF-κB and an increase in the expression of cell adhesion molecules in the lung tissue [4]. The addition of SS to human airway epithelial cell cultures A549 increased the activity of glutathione peroxidases and also inhibited both the formation of hydrogen peroxide and the activation of NF-κB. The authors believe that SS regulates the activity of NF-κB not only by increasing the activity of glutathione peroxidases (removing potential activators of NF-κB), but also, possibly, through the direct oxidation of critical sulfhydryl groups of this transcription factor.

When studying the effect of SS (200 μg/day for 8 weeks) on the ability of human blood lymphocytes to respond to stimulation with an alloantigen and turn into cytotoxic lymphocytes (T-killer cells), as well as on the activity of NK-cells, it was found that taking SS led to a 118% increase in cytotoxicity of the tumor mediated by cytotoxic lymphocytes, and an increase in the activity of natural killer cells by 82.3% compared to the baseline values. This, apparently, was associated with the ability of SS to enhance the expression of IL-2 receptors, and, consequently, with an increase in the rate of proliferation and differentiation of cells into cytotoxic ones [66].

The role of SS in protection against viral diseases of various etiologies has also been repeatedly shown. There is a negative correlation between the amount of SS and mortality in patients with hepatitis B associated with liver cancer [67]; in patients with hantavirus (“epidemic hemorrhagic fever”) with the introduction of oral SS, there was an 80% reduction in mortality [68]. There is evidence of the effect of selenite on the Ebola virus. Based on these data, a possible mechanism of the effect of selenite on the prevention and treatment of the SARS-CoV-2 coronavirus was suggested [49]. SARS-CoV-2 is an enveloped single-stranded RNA virus that targets cells expressing ACE2: airway epithelial cells, alveolar epithelial cells, vascular endothelial cells, and macrophages in the lungs, as well as myocardial and kidney cells, causing aggressive inflammatory reactions and respiratory and gastrointestinal pathogenic conditions. Selenite prevents thiol/disulfide exchange initiated by protein disulfide isomerases (PDI), which are redox enzymes that regulate thiol/disulfide balance in the interaction between SARS-CoV/CoV-2 spike proteins and ACE2 during the attachment of viral glycoproteins to host cell membranes, thereby limiting the ability of the virus to enter the cells [69].

Selenium in SS is a tetravalent cation capable of accepting two electrons and becoming divalent, thus it is an oxidizing agent and easily reacts with sulfhydryl groups in the active center of the viral protein disulfide isomerase, thus deactivating it. This leads to the viral hydrophobic spike losing its ability to interact with the disulfide groups of the proteins of the cell membrane and the virus cannot enter the cytoplasm of a healthy cell. The same mechanism underlies the action of oxidants with a pronounced antiviral activity: phenol, hydrogen peroxide, and hypochlorites (sodium hypochlorite is one of the main substances used for disinfection all over the world). However, these substances are applicable for the treatment of hospital rooms; instruments; and, in extreme cases, skin, mucous membranes, and wounds, and are not suitable for ingestion. The toxicity of SS is considered high, but LD50s vary greatly with the duration of SS doses. It was shown that the intravenous administration of SS at a dose of 500 μg/day is completely non-toxic [70], in addition, relatively high doses of SS (up to 2000 μg/day) are well tolerated by patients with peritonitis [3]. Thus, SS may be considered a promising agent for anticoagulant therapy and may reduce the risk of blood clots in COVID-19 patients.

## 3. Role of Organic Selenium Compounds in the Immune System and in Protection against COVID-19

The most famous and studied organic compounds of Se include selenomethionine (SLM), methylselenocysteine (MSC), methylseleninic acid (MSA), a representative of synthetic diselenides−ebselen, and many others. Organic forms of Se are metabolized in the body in different ways, which largely depends on their chemical structure, and thus they exhibit different mechanisms of action. For example, SLM and MSC can be metabolized to methylselenol (CH_3_SeH), selenocysteine (Sec), and selenide. Moreover, the formation of methylselenol from SLM requires several enzymatic reactions, while MSC is converted in one step [71,72].

MSC and Sec can be metabolized to inorganic Se, but some synthetic organoselenium compounds (for example, ebselenium) are unable to release Se from its organic part. It should be noted that organic forms of Se will promote the synthesis of selenoproteins only when they can be metabolized to selenide [72]. The undoubted advantages of organic selenium compounds are biocompatibility, easy digestibility, minimal toxicity, availability, and low cost. Thus, the high bioavailability and their absorption from food/supplements can reach 85–95%, while the absorption range of inorganic selenium is 40–50% [73,74]. All of these properties of organoselenium compounds stimulated a wide range of biomedical research involving them as promising therapeutic substances capable of modulating a wide range of related biological processes in the body, both in physiological and pathological conditions (Se deficiency, cancer, diabetes, and cardiomyopathy, as well as bacterial, mycotic, and viral diseases) and the possibility of developing new treatment strategies [75,76,77,78,79].

### 3.1. Role of Methylseleninic Acid in the Immune System and in Protection against COVID-19

**Figure 3:**
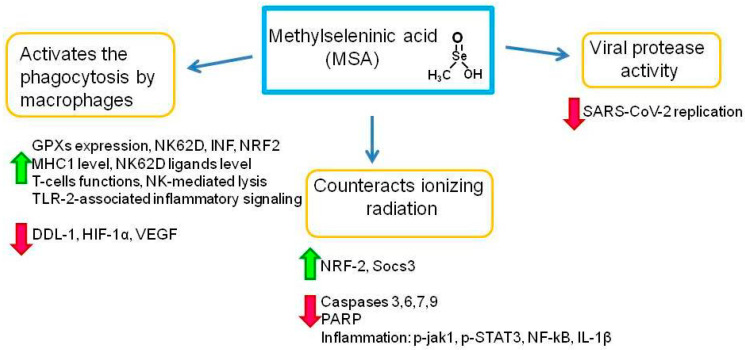
Role of methylseleninic acid in the regulation of the immune system and cytoprotections. GPX—glutathione peroxidases; NK62D—transcription factor; INF—interferon; NRF2—nuclear factor erythroid 2–related factor 2; MHC1—major histocompatibility complex (class I); TLR-2—toll-like receptor 2; DDL-1—DDHD domain-containing lipase 1; HIF-1α—hypoxia-inducible factor 1-alpha; VEGF—vascular endothelial growth factor; Socs3—suppressor of cytokine signaling 3; PARP—poly (ADP-ribose) polymerase; p-jak1—phosphorylated janus kinase 1; p-STAT3—phosphorylated signal transducer and activator of transcription 3; NF-κB—nuclear factor kappa-light-chain-enhancer of activated B cells; IL-1β—interleukin-1 beta. Expression enhancement is indicated by green arrows and suppression by red arrows.

Methylseleninic acid (MSA) (Figure 3), an organic monomethylated compound of Se, which is rapidly metabolized in the body to methylselenol (CH_3_SeH) in a simple stoichiometric manner immediately after entering cells, is easily recovered through both non-enzymatic and enzymatic reactions involving glutathione (GSH) and NADPH [72,80]. MSA is particularly advantageous in cell culture experiments, because it is stable in solution and provides rapid formation of methylselenol [81]. In addition to the mechanisms of direct anticancer action of MSA, this compound is able to prevent the development and spread of tumors indirectly, affecting the activity of immune cells, thereby exhibiting immunomodulatory properties. It has been shown that MSA affects the level of cytokines, the expression of histocompatibility proteins MHC1, enhances the functional activity of CD8 cytotoxic T-lymphocytes and NK cells, and activates the phagocytosis of tumor cells by macrophages [82]. Thus, it was shown that MSA (3 mg/kg body weight) inhibited tumor growth up to 61% compared with the control group, and this inhibition was associated with a decrease in the level of plasma (TNFα)/interleukin 6 (IL6), and an increased level of GPXs in blood [83].

Methylselenol, being a metabolite of MSA, affects innate immune responses: increased expression of NKG2D (natural killer group 2 member D) ligands, activation of NK cells, and increased expression of interferon (IFN). Transformed, infected, or stressed cells can prevent the immune system from functioning through the surface expression of NKG2D ligands, which are recognized by effector immune cells and are eliminated [84,85]. The number of different escape mechanisms used by viruses and non-transformed cells to defend against this form of immune recognition underscores the importance of the NKG2D system. The balanced expression of NKG2D ligands on the cell surface is considered an innate barrier against tumor development. MSA influences the immunogenicity of tumors by modulating various ligands of the NKG2D receptor, such as molecules associated with the MHC1 polypeptide, which leads to an increase in the responses of NK cells and some activated CD4^+^ T cells [85]. It is the Se metabolite methylselenol that modulates the expression of NKG2D ligands on the cell surface, regulating NKG2D ligands both at the transcriptional and posttranscriptional levels. Thus, these works show that the use of Se compounds that are metabolized to methylselenol, in particular MSA, can improve NKG2D-based immune therapy [86].

Complete or partial loss or suppression of the surface expression of MHC class I has been found in many tumors of various origins, which limited the corresponding antitumor immune responses and led to tumor progression [87,88]. Therefore, one of the main goals in the field of tumor immunology is the restoration and maintenance of MHC 1 expression in tumors, thereby increasing their immunogenicity. When treating B16F10 melanoma cells with different concentrations of MSA, it was observed that MSA-mediated changes in the redox metabolism of tumor cells resulted in an increase in MHC 1 levels on the cell surface. In addition, it was observed that MSA is able to partially mimic the signaling of interferon gamma (IFNg), to enhance the expression of STAT1, JAK1, IRF1, IRF5, IRF7, and IRF9.

In addition, MSA treatment led to the activation of the Nrf2 transcription factor and the genes involved in antioxidant protection. Under normal conditions, Nrf2 is bound to its inhibitor protein Keap1 and is labeled for proteasome degradation. Methylselenol modifies the redox residues of cysteine of Keap1, which leads to conformational changes and the release of Nrf2, which, in turn, can move into the nucleus and initiate the induction of transcription of its target proteins, one of which is NQO1 (NAD(P)H dehydrogenase [quinone]1). In addition to its role in antioxidant defense, NQO1 stabilizes p53 and thus prevents its proteasome degradation. In turn, p53 regulates the expression of TAP1 (transporter associated with antigen processing 1), which enhances the transport of peptides and the expression of MHC 1 peptides to the cell surface [88].

It has been shown that MSA stimulates NK-mediated lysis of A2780 ovarian cancer cells and enhances the cytolytic activity of T cells. Increased T cell function has been associated with decreased levels of PDL1 (programmed death-ligand 1), HIF-1α (hypoxia-inducible factor 1-alpha), and VEGF (vascular endothelial growth factor). It is known that the secretion of VEGF by tumor cells causes the endothelial cells to activate prostaglandin E2 (PGE2), which leads to the suppression of T-cell functions. Thus, MSA does not kill or inactivate immune cells at the doses required for anti-cancer treatment, but rather has an immunostimulatory effect, enhancing the T-cell-mediated destruction of tumor cells by inhibiting tumor-secreted PDL1 and VEGF [89].

It was found that MSA is able to attenuate the activation of the TLR2-associated inflammatory signaling pathway by reducing the expression of the caspase pathway proteins [90]. Thus, MSA exerted a protective effect against inflammatory lesions of the uterus in endometritis caused by *S. aureus* in rats. This was confirmed by the fact that the addition of MSA decreased the expression level of the inflammatory cytokines TNF-α and IL-6; the degree of phosphorylation of IκBα and NF-κB; and the suppression of the expression of caspases 3, 6, 7, and 9, as well as PARP polymerase. Thus, MSA supplementation may be a potential measure for the prevention and control of *S. aureus* endometritis.

In another work, the authors also demonstrated the antimicrobial and anti-inflammatory effects of MSA. It has been shown that MSA, most likely acting as a Se donor, exerts an antimicrobial effect, limiting the intracellular growth of *Mycobacterium tuberculosis* by activating c-Jun-mediated autophagy and LC3-associated phagocytosis of alveolar macrophages infected with Mycobacterium tuberculosis [91].

Ionizing radiation stimulated the development of the inflammatory response of the testes in rats by increasing the phosphorylated form of Janus kinase 1 (p-JAK1) and p-STAT3, which induced an increase in the expression levels of inflammatory markers NF-κB and IL-1β. In addition, radiation induced an increase in nitric oxide and malondialdehyde levels, followed by a decrease in glutathione (GSH) and superoxide dismutase (SOD) levels in the testes. MSA significantly counteracted the effects of radiation through the activation of nuclear factor Nrf2 and Socs3, and also contributed to the inhibition of testicular inflammation by reducing the levels of p-JAK1, p-STAT3, NF-κB, and IL-1β [92].

It has already been noted that MSA (a precursor of methylselenol) has a strong redox activity capable of leading to global modifications of any proteins containing thiols, including viral ones. The main viral protease Mpro SARS-CoV-2 is involved in the replication mechanism of coronavirus and, therefore, it is responsible for copying the genetic material and reproducing SARS-CoV-2 [54,93]. Given the critical importance of Mpro for viral replication, it represents one of the most promising molecular targets for inhibiting the multiplication of the SARS-CoV-2 virus. Given that the Cys145 Mpro residue of SARS-CoV-2 is a vital target for inhibition, it can be assumed that MSA or methylselenol may react with HS-Cys145-Mpro, resulting in a decrease in replication, transcription, and a shortened life cycle of SARS-CoV-2 [94]. A positive property of MSA in this case is also that it can accumulate in cells infected with SARS-CoV-2, thereby creating a certain “drug pool”. This is due to the fact that viral respiratory infections suppress antioxidant enzymes and induce enzymes that generate ROS, which leads to an increased production of ROS and oxidative stress and an increase in the accumulation of oxidation products, creating conditions in which the “reverse” reaction is assumed to occur and methylselenol is converted to methylselenic acid. In normal cells with lower levels of oxidants, volatile methylselenol is not oxidized to non-volatile methylselenic acid and thus is not retained by normal cells. The accumulated MSA, in turn, can inactivate SARS-CoV-2 Mpro in infected cells by modifying the Cys145 Mpro residue [79].

There are a number of works demonstrating the immunomodulatory abilities of MSA, including inhibition of the activity of the transcription factor NF-κB, which is an inflammatory marker and controls the expression of target genes for the immune response and apoptosis [95,96,97]. Activation of the NF-κB signaling pathway in various virus families increases viral replication and suppresses the apoptosis of a virus-infected cell. This signaling pathway also plays an important role in the progression of COVID19, which is associated with the occurrence of a “cytokine storm”, a reaction that ultimately leads to apoptotic death of lung cells and acute lung injury [98,99]. MSA stimulates alveolar macrophages, which are also able to phagocytose infected cells, and are found in large numbers in the lungs, which are the main target for SARS-CoV-2 attack [100].

### 3.2. Role of Selenomethionine in the Immune System and in Protection against COVID-19

**Figure 4:**
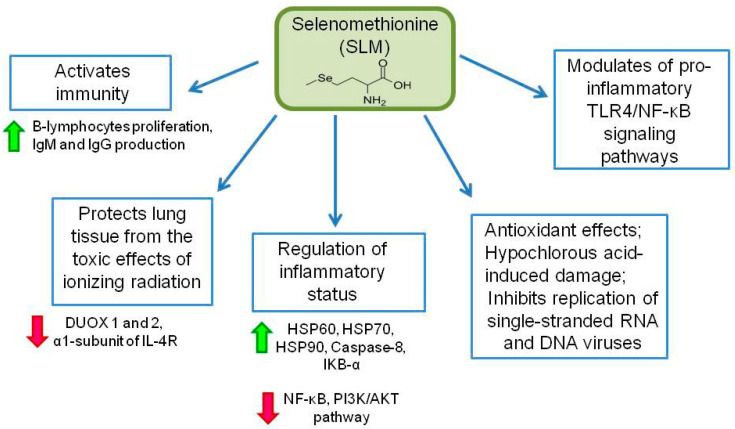
Role of selenomethionine in immune system activation. IgM and IgG—immunoglobulins M and G; DUOX 1 and 2—dual oxidase 1 and 2; IL-4R—interleukin-4 receptor; HSP60, 70 and 90—heat shock proteins 60, 70 and 90; IKB-α—nuclear factor of kappa light polypeptide gene enhancer in B-cells inhibitor, alpha; NF-κB—nuclear factor kappa-light-chain-enhancer of activated B cells; PI3K/AKT—PI3K-PKB/Akt pathway, protein kinase B (PKB or AKT) and phosphoinositide-3-kinase (PI3K); TLR4—toll-like receptor 4. Expression enhancement is indicated by green arrows and suppression by red arrows.

Selenomethionine (SLM) (Figure 4), like MSA, is a monomethylated form of a Se-containing compound, is a precursor of methylselenol and has a high redox activity. SLM is able to nonspecifically replace methionine in proteins, thereby endowing these proteins with an additional redox activity. Absorbed SLM is either directly metabolized to reactive forms of selenium or accumulates instead of methionine in body proteins. SLM metabolism, as well as its biological and pharmacological properties, differ from those of MSA, despite the similarity in their chemical structure. Proteins and enzymes containing SLM exhibit slightly different and actual additional properties compared to their unsubstituted counterparts. Amino acid substitution does not significantly change the structure of the protein, but it can affect the activity of the enzymes. As the CH3-Se group in SLM is more hydrophobic than the CH3-S group in methionine, access to the substrate can be impaired by changing the kinetic parameters. In general, these proteins and enzymes are also good and are often catalytic antioxidants [101]. As SLM is a powerful antioxidant capable of protecting cells from oxidative stress and maintaining redox homeostasis, it is able to exert its activity in all pathophysiological processes associated with oxidative stress, such as inflammation, microbial and viral infections, oncogenesis, coronary artery disease, neurodegenerative diseases, and many others.

The active work of the antioxidant system is especially important for immune cells, as, while performing their function, they often fall into a state of oxidative stress. The addition of 0.4 mg/kg Se from an organic source of SLM to food led to an increase in the productivity and responses of the immune system. It has been shown that SLM enhances the proliferation of B-lymphocytes in response to mitogenic stimulation, and also increases the production of IgM in vitro. Pharmacological levels of dietary Se increased the level of antigen-specific IgM and IgG in mice infected with sheep erythrocytes. Based on the results of the work, the authors suggest an important role of dietary Se, as well as SLM, as a source of Se, in maintaining optimal humoral immune function in vivo [102].

SLM, by modulating the activity of cells of the immune system, has anti-inflammatory properties [103]. Thus, SLM is able to reduce the effect of inflammation in various tissues and organs [103]. A study investigating the effect of organic Se (SLM) on lipopolysaccharide (LPS)-induced inflammation in chicken trachea showed that SLM supplementation enhances immune function and selenoprotein expression, and also reduces LPS-induced inflammation by inhibiting the NF-κB pathway [104,105]. Another study investigated LPS-induced inflammatory kidney damage in broilers. SLM has been demonstrated to have antioxidant and anti-inflammatory effects, and a potential molecular mechanism has been suggested. Histopathological observation demonstrates that SLM ameliorates the LPS-induced characteristic changes in renal inflammatory disease. In addition, SLM modulated the LPS-induced inhibition of the PI3K/AKT pathway, increased expression of caspase 8 and IκB-α, necroptosis marker genes (FADD, RIP1, RIP3, MLKL, and TNF-α), pro-inflammatory factors (NF-κB, PTGE, COX-2, iNOS, IL-1β, and IL-6) and overexpression of HSP60, HSP70, and HSP90 [106]. A similar study was conducted by Cui Y et al., where they showed that SLM improves LPS-induced intestinal immune dysfunction in chicken intestines. LPS supplementation has been shown to reduce selenoprotein expression and induce an inflammatory response, impaired control of cytokine production, decreased immunoglobulin levels, impaired heat shock protein expression, and decreased jejunal defensin expression levels. However, dietary SLM effectively reversed the aforementioned disorder, immune dysfunction caused by LPS [107]. SLM modulation of the pro-inflammatory TLR4/NF-κB signaling pathway and macrophage activity was described in the study of inflammation of the mammary gland of cattle (mastitis) induced by *E. coli*. SLM attenuated inflammation caused by *E. coli* by modulating the transmission pathway signaling Toll-like receptor 4 (TLR4)/NF-κB and stimulated macrophages, enhancing their migration and activity [108].

Work has been carried out to study the effect of levothyroxine (LT4) and SLM on systemic inflammation and the release of cytokines by monocytes and lymphocytes in patients with Hashimoto’s thyroiditis. Hashimoto’s thyroiditis is a common chronic autoimmune disease, a non-infectious inflammation in the thyroid gland in which the immune system aggressively targets thyroid cells. Although LT4 and SLM have been shown to affect different types of inflammatory cells, they have similar systemic anti-inflammatory effects in women with Hashimoto’s thyroiditis—lead to a decrease in antibody titers to thyroid peroxidase. This fact may have a clinical advantage in the prevention and treatment of Hashimoto’s thyroiditis with two agents at once [109]. The immunomodulatory effects of Se were additionally considered in terms of modulating the secretion of interferon-induced chemokines, compounds involved in the progression of several autoimmune endocrine diseases (CXCL9, CXCL10 and CXCL11). It has been shown, that the administration of SLM to euthyroid women with autoimmune thyroid disease (AITD) reduced serum levels of CXCL9, CXCL10 and CXCL11 [110].

It has been shown that SLM protects lung tissue from the toxic effects of ionizing radiation by reducing the expression of the DUOX1 and 2 genes (Dual Oxidase) and the levels of the α-1 subunit of the IL-4 receptor [39]. And as you know, the protective role in relation to the lungs is of great importance in COVID-19, since the target organ of SARS-CoV-2 is the lung tissue. During inflammation, in addition to ROS, myeloperoxidase (MPO), activated immune cells, hypochlorous acid (HOCl) is released in large quantities, which, in addition to its antimicrobial effect, can cause damage to host tissues. Vascular smooth muscle cells are an important target for HOCl. For this reason, researchers are looking for therapeutic approaches to reduce the formation of HOCl. Selenium-containing compounds in this case are attractive therapeutic agents, since they quickly react with HOCl through selenium oxide, and easily undergo catalytic transformations due to their redox potential. It has been shown that SLM can modulate HOCl-induced damage to human coronary artery smooth muscle cells and act as a HOCl scavenger to reduce HOCl-induced cellular damage in inflammatory foci [111].

In addition, it has been shown that SLM can inhibit single-stranded RNA viruses such as Coxsackie virus, hepatitis C virus (HCV) and human immunodeficiency virus (HIV) at a concentration of 50 μM, as observed in in vitro studies [40]. SLM has been reported to inhibit porcine circovirus type 2 (PCV2) in vitro in a concentration-dependent manner ranging from 2 to 16 μM. PCV2 is a single-stranded DNA virus associated with several complications including porcine dermatitis nephropathy syndrome (PDNS), post-weaning multisystem wasting syndrome (PMWS), porcine respiratory disease complex (PRDC), type A2 congenital tremor (CT), and reproductive obstruction pregnant sows [101]. In these studies, attention is drawn to the large differences in the concentrations of SLM required for antiviral activity against single-stranded RNA and DNA viruses, which may indicate some selectivity with respect to the type of viruses. This assumption requires further research.

There are interesting data on the effect of SLM supplementation in vaccination against influenza virus. The addition of SLM (92–98 μg/L) to people with minimally low plasma Se levels enhanced the cellular immune response to live polio vaccine and viral clearance, but did not directly affect the antibody titer after influenza vaccination. The addition of SLM (100 μg/day) to people with slightly low Se status resulted in some positive effect on cellular immunity, with significantly higher T cell proliferation in the group. However, higher supplementation doses (200 μg/day SLM in yeast matrix) resulted in a decrease in CD8 lymphocyte counts, while other forms of Se (e.g., sodium selenite) did not affect the immune response to influenza vaccination [112].

The antiviral potential of SLM has been demonstrated in preclinical studies of the efficacy and safety of SSV-003, a potent herbal antiviral drug. SLM is combined with curcumin, vitamin C, vitamin K2-7, and zinc in SSV-003, an antiviral drug with activity against influenza A (H1N1) virus and human beta coronavirus (ATCC^®^ VR1558™). Studies of acute toxicity and therapeutic effects were carried out using cell lines MDCK and HCT-8 and in Balb/c mice infected with influenza A virus in vivo. The results showed that the composition of SSV-003 showed a strong antiviral activity against both selected virus strains. Its IC50 was significantly lower than that of ribavirin against influenza A-H1N1-VR219, with no cytopathic effect. In mice, a dose-dependent significant increase in the number of CD4+ and CD8+ lymphocytes, as well as the levels of IgG and IgM was observed. No acute oral toxicity or morbidity was observed in the study [113].

In the search for potential antiviral drugs for the treatment of the 2019 novel coronavirus based on DFT calculations and molecular docking, SLM, among others, has been proposed as a potential antiviral candidate for the treatment of COVID-19 [114]. Based on the above, it can be assumed that SLM can be used in the therapy and prevention of viral diseases, including COVID-19 due to its multiple properties—the ability to attack the components of the virus, act as an antioxidant and redox modulator, and modulate and strengthen the immune host system.

### 3.3. Role of Se-Methylselenocysteine in the Immune System and in Protection against COVID-19

**Figure 5:**
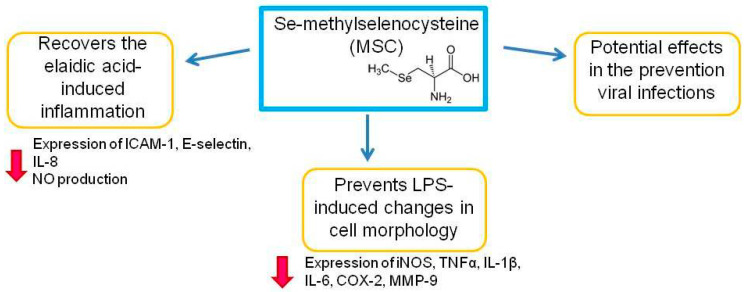
The role of Se-methylselenocysteine in the immune system activation and inhibition of inflammation. ICAM-1—inter-cellular adhesion molecule 1; IL-8—interleukine-8; NO—nitric oxide; iNOS—inducible nitric oxide synthase; TNFα—tumor necrosis factor alpha; IL-1β—interleukin-1 beta; IL-6—interleukine-6; COX-2—cyclooxygenase 2; MMP-9—matrix metallopeptidase 9. Suppression of the gene expression is indicated by red arrows.

Se-methylselenocysteine (MSC) (Figure 5), a derivative of the methylation of selenocysteine, is a natural monomethylated selenoamino acid, an analogue of S-methylcysteine, in which the sulfur atom is replaced by a selenium atom. MSC is chemically less reactive than other organic selenium compounds [115,116] and is considered a good source of dietary Se because after ingestion, it is relatively stable as a free amino acid and is difficult to incorporate into proteins. In mammals, MSC is metabolized by kynurenine aminotransferase 1 (KYAT1), either through transamination to form methylselenopyruvate or through a beta-elimination reaction to form monomethylselenol [117]. Methylselenol is volatile and has exceptional nucleophilic properties [118].

The authors investigated the effect of MSC supplementation on elaidic acid-induced inflammation of human arterial endothelial cells (HAEC). Elaidic acid is a component of industrial trans fatty acids (I-TFA) and promotes the expression of inflammatory cytokines in the body. In addition, published clinical studies have shown that I-TFAs are closely associated with endothelial cell damage, cardiovascular disease, and insulin resistance. Elaidic acid causes endothelial cell dysfunction by increasing the levels of inflammatory cytokines such as intercellular adhesion molecule 1 (ICAM-1), and contributing to endothelial cell damage by increasing NF-kB expression and nitric oxide (NO) levels. When analyzing the viability of HAEC cells, the authors saw that elaidic acid significantly reduced cell viability, and MSC effectively reversed this process, especially at a concentration of 200 μmol/L with 24-h incubation. In addition, the same concentration of MSC prevented elaidic acid-induced apoptosis of HAEC cells. At the same time, a decrease in the expression of ICAM-1, E-selectin, IL-8, and NO production in HAEC induced by elaidic acid was observed. Based on these results, the authors concluded that MSC regulated the expression of ICAM-1, E-selectin, and IL-8 to protect against elaidic acid-induced inflammation in HEAC [119].

The anti-inflammatory activity of MSC was investigated using the example of RAW 264.7 murine macrophages activated by lipopolysaccharides (LPS). It is known that macrophages play a central role in the immunopathological events accompanying inflammation. They perform a protective function due to the phagocytosis of external pathogens and oncotransformed cells. However, overactive immune responses can lead to an overproduction of proinflammatory cytokines by macrophages such as tumor necrosis factor-α (TNF-α); interleukins (IL) -1beta, IL-6, and IL-12; and other inflammatory mediators such as oxide nitrogen (NO), matrix metalloproteinase-9 (MMP-9), and prostaglandin E2 (PGE2). These neurotransmitters can induce a sustained and excessive inflammatory response that leads to redness, swelling, pain, and even loss of function of the affected tissues. MSC suppressed the expression of RNA iNOS, TNF-α, IL-1β, IL-6, COX-2, and MMP-9, as well as the release of NO, TNF-α, IL-6, IL-12p70, COX-2, and PGE2 from LPS-activated macrophages RAW264.7, depending on the concentration. Moreover, MSC prevented LPS-induced changes in cell morphology. At the same time, a rather wide range of MSC concentrations (from 50 to 200 μM) showed an anti-inflammatory effect close to that of 20 μM dexamethasone. The authors demonstrated that MSC effectively inhibited the activation of RAW 264.7 macrophages induced by LPS, and suggested that MSC could be a potential functional nutritional component for the prevention or treatment of inflammatory diseases [120].

MSC has demonstrated potential effects in the prevention and treatment of human diseases such as cancer, endometritis, Alzheimer’s disease, and various other pathological processes associated with inflammation. A feature of MSC is that this Se compound is less toxic than other organic Se compounds or inorganic Se. Unfortunately, we did not find any work related to the antiviral activity of MSC. Perhaps due to the antioxidant, anti-inflammatory properties described above, and the ability to act as a source of Se and selenoproteins in the body, MSC has potential effects in the prevention and control of viral diseases, but such studies are not presented in the literature.

## 4. The Mechanism of Antiviral Action of Selenium Mediated through Selenoproteins and Selenium Nanoparticles

**Figure 6:**
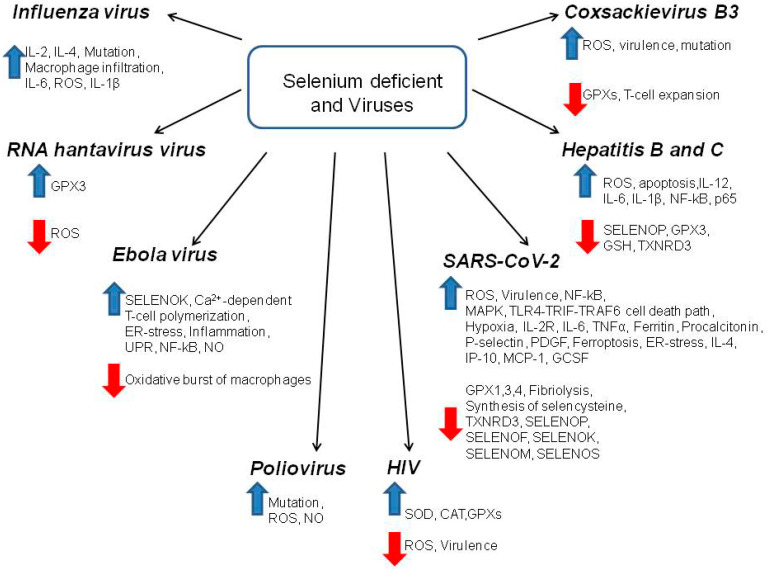
The relationship of Se deficiency with the mechanisms and pathogenicity of viral infections. IL-2, 4, 6, 12—interleukins -2, 4, 6, 12; IL-1β—interleukin-1 beta; ROS—reactive oxygen species; GPX1, 3, 4—glutathione peroxidases 1, 3, 4; SELENOK, SELENOF, SELENOM, SELENOS, SELENOP—selenoptoteins K, F, M, S, P; ER-stress—endoplasmic reticulum stress; UPR—unfolded protein response; NF-κB—nuclear factor kappa-light-chain-enhancer of activated B cells; NO—nitric oxide; SOD—superoxide dismutase; CAT—catalase; MAPK—mitogen-activated protein kinase; IL-2R—interleukine-2 receptor; TNFα—tumor necrosis factor alpha; PDGF—platelet-derived growth factor; IP-10—interferon gamma-induced protein 10; MCP-1—monocyte chemoattractant protein 1; GCSF—granulocyte colony-stimulating factor; TXNRD 1, 3—thioredoxin reductase 1, 2; GSH—glutathione; p65—nuclear factor NF-kappa-B p65 subunit. Expression enhancement is indicated by green arrows and suppression by red arrows.

The key event in cell infection with viruses is ROS production and oxidative stress [31]. Selenium, through the regulation of the expression and activity of selenoproteins, is able to exert an antiviral effect through the mechanism of cell protection from oxidative stress (Figure 6). It has been shown that in mice with knockdown of the gene encoding the SELENOK, decreased viral clearance and elevated viral titres were observed in the brain when they were infected with West Nile virus, as well as decreased Ca^2+^ flux in the ER of T-lymphocytes, macrophages, and neutrophils [34]. Reticulum stress, oxidative stress, and inflammation are closely related processes, i.e., ER stress can lead to inflammation, just as inflammation can lead to UPR and ER stress [121]. An overproduction of free radicals in response to infection in immune cells triggers oxidative stress and ROS (reactive nitrogen species), accumulating in excessive concentrations, which cease to perform their signaling function and can activate pro-inflammatory signaling pathways involving IL-1 β, IL-6, and TNF-α, and activating NF-κB signaling pathways [122,123].

The presence of sequences encoding selenoproteins in the genome of viruses themselves is also evidence of the protective effect of selenoproteins against viral infections. For example, Ebola Zaire, the hemorrhagic fever virus, has several SECIS-elements in its genome, and the Ebola Reston strain lacking these elements is not pathogenic for humans [124]. Similarly, the HIV-1 genome has also been identified to have potential selenoproteins that can be expressed by ribosomal frameshifting and termination suppression [125]. In addition, the presence of Se-dependent GPX modules was shown in the sequences of HIV-1, HIV-2, hepatitis C virus, coxsackie virus, and measles virus. It is assumed that the presence of selenoprotein-like GPX sequences in the viral genome is necessary as a defense mechanism of the virus against the action of free radicals of the immune system of the infected organism [126]. These selenoproteins are a necessary element of protection not only for host cells, but also for the viruses themselves.

For COVID-19, a significantly lower mortality rate of infected people is shown in regions with a normal supply of Se, compared to selenium-deficient regions. In addition, normal SELENOP and zinc levels correlate with high chances of survival for those infected with the coronavirus [23,127]. Normal serum Se and SELENOP levels should be maintained at 45.7–131.6 μg/mL and 2.56–6.63 mg/L, respectively, and in COVID-19 patients, these levels are reduced by 44.4% and 39.6%, respectively [29]. An in vitro model has shown an increase in ROS production upon infection with SARS-CoV through the activation of Nf-kB by viral protease. Studies by Korean and German scientists have shown a clear correlation between mortality in patients with COVID-19 and low Se levels and SELENOP [25,29]. It has been shown that SARS-CoV-2 does not reduce the expression level of TXNRD1 and TXNRD2, but suppresses the level of TXNRD3 by 36.9% [128]. SARS-CoV-2 enhances the production of ROS in the mitochondria, which accelerates the replication of the viral particles themselves, and the use of the antioxidants mitoquinol and N-acetylcysteine significantly inhibits the replication process [129]. GPX4 is the only protein from this family of peroxidases located in the mitochondria, and it protects cells from death due to ferroptosis. At the same time, with COVID-19, leukopenia is observed, which occurs due to ferroptosis in leukocytes due to the suppression of GPX4 by the virus [130].

When infected with SARS-CoV-2, the expression of selenoproteins residing in the endoplasmic reticulum is impaired, which leads to ER stress. SARS-CoV-2 leads to a decrease in the expression of SELENOF, SELENOM, SELENOK, and SELENOS, which increases the concentration of misfolded proteins in the ER. SELENOF expression has been shown to be the most affected by SARS-CoV-2 infection (75.9% decreases). SELENOF may be targeted for proteolysis by the SARS-CoV-2 main protease Mpro, because the SELENOF protein contains a sequence (TVLQ/AVSA) that is almost identical to a known viral Mpro cleavage site (TVLQ/AVGA). This dual targeting of SELENOF by SARS-CoV-2 at both the mRNA and protein levels suggests that the disruption of the SELENOF function may be particularly important for SARS-CoV-2 replication [131]. Infection of Vero E6 cells with the SARS-CoV-2 virus significantly reduced the expression of selenoproteins GPX4, TXNRD3, SELENOS, SELENOK, SELENOF, and SELENOM, while the level of the inflammatory cytokine IL-6 increased against this background.

MSRB1 is the only selenocysteine-containing member of the methionine sulfoxide reductase (MSR) protein family. This selenoprotein has been shown to play an important role in the regulation of actin polymerization during cellular activation, which contributes to functions such as phagocytosis and cytokine secretion [132]. Thus, macrophages use the redox system during cellular activation, stimulating the expression and activity of MSRB1 as part of the innate immunity. Subsequent research has shown that MSRB1 controls immune responses by promoting the expression of anti-inflammatory cytokines in macrophages [133]. In general, it remains to be determined how the regulated expression of MSRB1 in humans affects infection by this bacterium or other pathogens that replicate in the macrophages, but this selenoprotein appears to be very important for innate immune responses.

**Figure 7:**
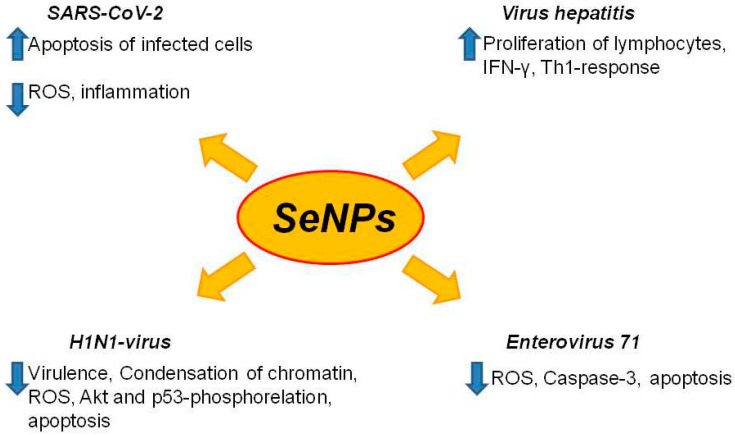
Known effects of selenium nanoparticles on viral infections. SeNPs—selenium nanoparticles; ROS—reactive oxygen species; Akt—Akt/protein kinase B; p53—transformation-related protein 53; IFN-γ—interferon gamma; Th1-response—Th1-type cytokines. Blue arrows pointing up indicate an increase in gene expression, arrows pointing down indicate a decrease in gene expression.

In recent years, approaches have appeared for the treatment of viral infections using selenium nanoparticles (SeNP), which, among other things, are doped with various active compounds to increase their effectiveness (Figure 7). Modern nanotechnologies make it possible to obtain SeNP of various shapes and sizes, including in the form of nanowires, which determines their properties and efficiency. It has been shown that spherical SeNP (35.6 ± 4.3 nm) effectively suppresses oxidative stress and apoptosis induction in C2C12 and promoting cell differentiation [134]. At the same time, selenium nanowires with a size of 10–20 µm in size with an aspect ratio of 50–100 have an extremely high antibacterial potential against a number of strains of multidrug-resistant Staphylococcus aureus [135]. SeNP are able to suppress oxidative stress, induce apoptosis of infected cells, and reduce the percentage of dying lung cells, while exerting minimal cytotoxic effects on healthy cells. SeNP doped with oseltamivir prevent the H1N1 influenza virus from binding to the host cell by inhibiting hemagglutinin and neuraminidase activity [36], as well as suppressing virus-induced chromatin condensation and DNA fragmentation, ROS production, and phosphorylation of Akt and p53, thereby having an anti-apoptotic effect [136]. Enterovirus 71 (EV71) can cause very severe symptoms and can kill infants and children under 6 years of age. There are currently no effective treatments for this viral infection, but there are studies that show an increase in the antiviral drug oseltamivir in combination with SeNP, which leads to the suppression of caspase-3 and ROS in the host cell [137].

Selenium nanoparticles have been shown to be highly effective in preventing hepatitis virus infection. When an antigen to the hepatitis virus was injected simultaneously with SeNP, activation of lymphocyte proliferation and total antibody responses was observed, and more importantly, an increased IFN-γ expression and induction of Th1 response. Thus, SeNP are capable of polarizing the immune response and increasing the effectiveness of the vaccine [138]. In general, SeNP, in view of the pleiotropy of Se itself, may be the most effective at combating SARS-CoV-2, as studies on both healthy cells and cancer have shown that SeNP is able to activate oxidative stress and ER stress in damaged cells [32,38].

## 5. Pharmacological and Toxicological Properties of the Selenium Compounds

Summarizing the above information, we can briefly describe the pharmacological and toxicological properties of selenium-containing compounds, which are the focus of this review. The pharmacological properties of the Se-containing compounds are manifested primarily through specific and nonspecific modulation of the activation of antioxidant pathways, as well as through the inhibitory effects of some selenium-containing proteins. When Se is administered to experimental animals at a nutritional dose (0.1 ppm), its physiological functions are believed to be mediated primarily by selenoproteins such as TXNRD3 and glutathione peroxidases. At high doses of Se (5 ppm), its toxicity is mediated by selenometabolites. If Se is administered for prophylactic anti-carcinogenic purposes at intermediate doses (1–3 ppm), it is not clear whether Se exerts its prophylactic action through selenometabolites, selenoproteins, or a combination of both [97].

It is known that some organic Se-containing compounds can exhibit a strong electrophilic activity, forming selenenyl sulfide bonds with cysteinyl residues of non-protein and protein thiols, while oxidizing them and forming intramolecular disulfide bonds that change the active state of proteins, thus inhibiting them. Such Se-containing compounds with pro-oxidant properties can disrupt mitochondrial homeostasis and induce cell death through apoptosis. It is due to these abilities that organic selenium compounds exhibit antimicrobial and antiviral properties, oxidizing important thiol-containing proteins of viruses, bacteria, and fungi [72,79].

Organic forms of Se are metabolized in the body in different ways, which largely depends on their chemical structure, and they thus exhibit different mechanisms of action. For example, SLM and MSC can be metabolized to methylselenol (CH_3_SeH), Sec, and selenide. Moreover, the formation of methylselenol from SLM requires several enzymatic reactions, while MSC is converted in one step [71,72]. Methylselenol is a key metabolite with an anticancer activity. However, oncological therapy requires its production in situ or, alternatively, the use of organic selenium compounds precursors due to the high reactivity and volatility of this molecule [94,97]. MSC Sec can be metabolized to inorganic selenium, but some synthetic organoselenium compounds (for example, ebselen) are not able to release selenium from its organic part. It should be noted that organic forms of selenium will promote the synthesis of selenoproteins only when they can be metabolized to selenide [72]. The undoubted advantages of organic selenium compounds are also biocompatibility, easy digestibility, minimal toxicity, availability, low cost. Organic selenium compounds are highly bioavailable and their absorption from food/supplements can be as high as 85–95%, while the absorption range of inorganic selenium is 40–50% [74].

Thus, orally administered of SS LD50 values range from 3 to 12 mg Se/kg body weight in rats [139,140], and from 7 to 22 mg/kg [141] in mice. With intraperitoneal administration, SS LD50 values range from 3.25 to 3.50 mg/kg of SS (for rats) [142]. LD50 for rabbits and cats is 1.5–3.0 mg/kg selenium, regardless of the route of administration [143]. The acute toxicity of selenium compounds is directly affected by their water solubility [140]—highly soluble sodium selenite was seven times more toxic than the less soluble selenourea compound and nine hundred times more toxic than insoluble elemental selenium. The toxicity of SS is considered high, but LD50s vary greatly with the duration of SS doses. It was shown that the intravenous administration of SS at a dose of 0.5 mg/day was completely non-toxic [70]; in addition, relatively high doses of SS (up to 2 mg/day) are well tolerated by patients with peritonitis [3]. Thus, SS may be considered a promising agent for anticoagulant therapy and may reduce the risk of blood clots in COVID-19 patients.

As mentioned above, Se-containing compounds act through the regulation of selenoprotein activity [94] and through the activation of the Ca^2+^ signaling system [4]. The pharmacology of the action of SeNPs lies in their endocytosis into cells and, probably, their inclusion in the metabolism. The size and shape of the SeNP allows for effective delivery of Se to cells and tissues, avoiding the toxic effects created by treatment with other Se-containing agents [144]. Compared to selenomethionine, SeNP shows 3.5 times lower toxicity, while compared to SS, SeNP shows a seven-fold lower acute toxicity (LD50 113 mg/kg for SeNP and 15 mg/kg for SS) [145]. Studies of liver enzymes in animals treated with synthetic SeNP showed that the use of nanoparticles at concentrations of 2.5, 5, and 10 mg/kg does not cause impairment of liver function and its key enzymes—alanine amino transferase, aspartate aminotransferase, alkaline phosphatase, creatine phosphokinase, and blood urea nitrogen. General body health indicators (hemoglobin, hematocrit, bilirubin, cholesterol, triglyceride, white blood cell counts, red blood cell counts, and platelet counts) also do not change after exposure to this wide range of SeNP doses. The use of 2.5 mg kg^−1^ selenium dioxide causes a violation of all of these indicators, as well as the manifestation of clinical symptoms of poisoning, including changes such as a decrease in skin color and a decrease in mobility, appetite, and body weight, leading to high mortality in laboratory animals [146].

## 6. Discussion

This review contains the latest data on the role of selenium-containing compounds of an inorganic and organic nature, as well as selenoproteins and selenium nanoparticles in the regulation of diseases of the immune system of various etiologies. Viral infections are a serious public health problem in many parts of the world due to their widespread prevalence, the large number of pathogenic viruses, and significant socio-economic damage, especially due to the recently emerging COVID-19 infection. Understanding the mechanisms of infection of the body with viruses, the characteristics of the course of the disease, and the relationship with individual vital trace elements will contribute to the development of more effective ways to resist viral infections.

The trace element Se is strongly involved in the regulation of the immune response, oxidative stress, and chronic inflammatory processes. The lack of selenium leads to an increase in the body’s sensitivity to a number of viral infections due to the effect on various intracellular mechanisms of both the virus itself and the host cell [1,2,3,4,5,6,7,8,9,10,11,12,13,14,15,16,17,18,19,20,21,22,23,24,25,26,27,28,29,30,31,32,33,34,35,36,37,38,39,40,147,148]. Adequate levels of bioavailable selenium are functionally important for several aspects of human biology, including the central nervous system, male reproductive biology, endocrine system, muscle function, cardiovascular system, and immunity [6,149]. Many pathological conditions involving the immune system can be influenced by a person’s selenium status, which can be influenced by several factors, such as the levels and forms of selenium ingested, conversion of selenium compounds to metabolites, and genetic characteristics. The predominant form of selenium that enters the human body is selenomethionine. Se is metabolized into various low molecular weight Se compounds, including those that can exert biological effects through redox reactions that can affect various cellular processes [150,151]. These bioactive metabolites include hydrogen selenide and sodium selenite and methylated selenium compounds—methylseleninic acid, selenomethionine, and methylselenocysteine [47].

In this review, based on the data available to date, including those obtained in our laboratory, some studies on the molecular mechanisms underlying the effect of selenium and selenium-containing compounds on the immune system are presented. Since Se can affect so many cellular functions, it is very difficult to isolate the many different pathways and individual molecules regulated by this trace element. Therefore, we have limited ourselves to the most common and well-studied Se-containing agents.

Among the inorganic forms of Se, the most studied is SS, which is involved in the differentiation of T-lymphocytes and enhances the expression of pro-inflammatory cytokines, VEGF, and NF-kB [64,65]. Thus, SS can prevent thiol-disulfide metabolism through disulfide isomerases, and is also involved in defense mechanisms against a number of viral infections—hepatitis B, hantavirus, Ebola virus, and SARS-COV-2 [66,67,68,69].

The most famous and studied organic compounds of Se include selenomethionine (SLM), methylselenocysteine (MSC), and methylseleninic acid (MSA). MSA is involved in the activation of macrophage phagocytosis, as well as the functioning of T cells, Nrf-2, and NK-mediated lysis [88]. MSA increases the expression of glutathione peroxidases in the blood [83], as well as the level NKG2D, INF, and MHC1, and modulates the activity of various NKG2D ligands [84,85,86].

MSA participates in the activation of the TLR-2-associated inflammatory signaling pathway through decreasing the expression of DDL1, HIF-1α, and VEGF [90]. MSA counteracts radiation through activation of Nrf-2 and Socs3, and at the same time, MSA reduces inflammatory processes by decreasing expression levels of p-JAK1; p-STAT3; NF-kB; IL-1β; caspases 3, 6, 7, and 9; and PARP polymerase. MSA has a pronounced antiviral activity, especially against the SARS-COV-2 virus—reduces its replication through reaction with protease HS-Cys-145-Mpro [54,93,94]. SLM enhances the proliferation of B-lymphocytes and the production of IgM and IgG [102,103]. In addition, it reduces LPS-induced inflammation by inhibiting the NF-kB pathway, by enhancing the expression of selenoproteins, caspase 8, IkB-α, necroptosis markers (FADD, RIP1, RIP3, MLKL, and TNF-α), pro-inflammatory factors (NF-kB, PTGE, COX-2, iNOS, IL-1β, and IL-6), HSP60, HSP 70, and HSP 90. In addition, SLM modulates LPS-induced inhibition of the PI3K/AKT pathway and pro-inflammatory TLR4/NF-kb signaling pathway [104,105,106,107,108].

In combination with levothyroxine, SLM enhances the anti-inflammatory effect in chronic autoimmune disease, such as Hashimoto’s thyroiditis [109,110]. SLM also protects lung tissues from the toxic effects of ionizing radiation by decreasing the expression of the DUOX 1 and 2, the α-1 subunit of the IL-4 receptor. SLM can modulate HOCl-induced damage, and inhibits single-stranded RNA and DNA viruses, including SARS-COV-2 [40,101,111,112,113,114]. MSC recovers the consequences of elaidic acid-induced inflammation by decreasing the expression of ICAM-1, E-selectin, IL-8, and NO production [119]. In addition, it prevents LPS-induced changes in cell morphology by decreasing the expression of iNOS, TNF-α, IL-1β, IL-6, COX-2, and MMP-9 [120]. Perhaps due to the antioxidant and anti-inflammatory properties described above and the ability to act as a source of Se and selenoproteins in the body, MSC has potential effects in the prevention and control of viral diseases, but such studies are not presented in the literature.

The importance of adequate dietary Se levels and its effective incorporation into selenoproteins for immunity has been demonstrated in cell culture and animal models. Among the 25 known human selenoproteins to date, some of them play an essential role in maintaining the immune function in the body. These include the following selenoproteins: SELENOP, SELENOK, glutathione peroxidase, thioredoxin reductase, and MSRB1 [34,121,122,123,124,125,126,127,128,129,130]. In recent years, approaches have appeared for the treatment of viral infections using Se nanoparticles (SeNP), which have been shown to be effective in preventing viral infections such as H1N1 influenza virus [36], Enterovirus 71 [136,137], hepatitis virus infection [138], and SARS-CoV-2 [32,38].

Thus, the data presented in this review provide a new understanding of the effect of Se, selenoproteins, and common Se-containing compounds on human immunobiology. In addition, special attention is paid to innovative developments, with the example of SeNP, which provide a better understanding of the molecular mechanisms underlying these effects.

## 7. Conclusions

In this review, the available studies of the molecular mechanisms underlying the action of selenium and selenium-containing compounds of various natures on the immune system are carried out, and their antiviral properties are studied. Much attention is paid to the latest research on the role of these compounds in protection against coronavirus infection (COVID-19), which is an extremely relevant and serious problem at the present time. The given data allow for comparing the effect of each of the selenium compounds in the regulation of the immune system, depending on the nature, physicochemical properties, and pharmacological and therapeutic actions of these compounds.

## Data Availability

The data presented in this study are available upon request from the corresponding authors.

## Abbreviations

ACE2	Angiotensin converting enzyme 2
CD4+T cells	Cluster of differentiation 4 T helper cells
CXCL 9, 10, 11	Chemokines 9, 10, 11
DFT	Density functional theory
FADD	Fas-associated protein with death domain
GPX4	Glutathione peroxidase 4
HSP 60, 70,90	Heat shock protein 60, 70,90
IL	Interleukin
iNOS	Inducible nitric oxide synthase
IRF	Interferon
JAK1	Janus Kinase 1
LAP	LC3-associated phagocytosis
MHCI	major histocompatibility complex
MLKL	Mixed lineage kinase domain like pseudokinase
MMP-9	Matrix metallopeptidase 9
NF-κB	Nuclear factor kappa-light-chain-enhancer of activated B cells
NK-cells	Natural killer cells
NO	Nitric oxide
NRF2	Nuclear factor erythroid-derived 2-like 2
P53	Tumor protein P53
PARP	Poly (ADP-ribose) polymerase
PI3K/AKT/mTOR pathway	Phosphatidylinositol 3-kinases/lpha serine/threonine-protein kinase/mammalian target of rapamycin
ROS	Reactive oxygen species
Se	Selenium
Sec	Selenocysteine
SELENO	Selenoprotein
STAT1	Signal transducer and activator of transcription 1
TLR2,4	Toll-like receptor 2,4
TNFα	Tumor necrosis factor α
TXNRD1,2,3	Thioredoxin reductase 1,2,3
